# Transactions T. S. M. A.

**Published:** 1887-09

**Authors:** 


### BIBLIOGRAPHY.

TRANSACTIONS TEXAS STATE MEDICAL ASSOCIATION FOR 1887. [19th Annual Convention, Austin, Texas, April, 1887. Printed for the Association by Eugene Von Boeckmann, Austin, Texas. F. E. Daniel, R. M. Swearingen and J. W. McLaughlin, Publishing Committee. F. E. Daniel, Secretary.]

This is a volume of 440 pages, in paper cover, having a cut of the new capitol of Texas on the cover, and an electrotype of Dr. S. R. Burroughs, President of the Association, on the title page.

The first 92 pages contain the officers and committees, and the Secretary's Minutes of the four days proceedings. This is followed by the President's address—a document which must be read to be properly appreciated.

Then follows the Section work, beginning with the report of the Chairman of each section.

On Practice, and Materia Medica, etc., the Chairman's report, a paper on "Fevers," by Dr. J. F. Y. Paine, Galveston, is an able and exhaustive production. The Doctor confines himself to continued fevers, and says he has drawn mainly from his own store of theories and experiences. He makes no attempt to offer a rationale of the phenomena of all fevers,—but says that "the ancient notion that febrile paroxysm is a salutary effort of nature to free herself from some oppressive load, or morbific matter, harmonizes with the germ theory of to-day." The main argument is to show that all continued asthenic fevers are not typhoid (a very popular falacy), and that intestinal lesions are not absolutely pathognomonic of typhoid fever. A most interesting and instructive paper, well worthy of a permanent place in the literature of the subject. Dr. Paine will be remembered as the late able and popular Professor of Materia Medica, etc., in the University of Louisiana, of which institution he is an *alumnus* of '61.

"Babies and their Troubles," by Dr. C. L. Gwyn of Galveston, is a short and sensible paper, in which the author argues that the great mortality of infants is largely due to errors in feeding; and emphasizes the precept, that a child should not be fed until the period of dentition, provided the mother is healthy, and the supply of nourishment sufficient. He lays down some good hygienic rules which, if followed, would result in saving many a weakly infant.

Dr. E. Goldman of Galveston, has a sensible paper on mental alienation—which he has observed to result from intermittent (malarial) fever.

Dr. O. L. Williams gives some hints on the use of antipyrin; well worth reading.

In his paper, entitled "Irritation of the Spinal Cord," Dr. J. W. Carhart of Lampasas suggests that certain spinal trouble *may be* due to the negligent manner in which corn meal is sometimes prepared in this country. He points out that frequently there is "smut" (diseased grain) at the end of the "ear" of corn, and that it is not separated before the corn is ground, and draws a parallel between its action and that of ergot, (diseased rye) which "is known to produce anæmia." He says:—"It is now a well recognized fact that irritation of the cord is the result, not of inflammation, but of anæmia; and hence, is accounted for, the prevalence of sore shins and spinal irritation in Texas."

An excellent paper by Dr. E. J. Ward of Waxahachie, is entitled "Typho-Malarial Fever;" it is the most complete paper on the subject we have seen. The author reviews the literature on the subject in a manner as pleasing as it is thorough; pays a tribute to Woodward, and defends the name, and the existence of the disease. "That such a fever does exist in localities where malarial and typhoid fevers are endemic, rests upon irrefragable testimony;" and again: "But if it can be shown that the disease is a hybrid, presenting the clinical features of typhoid and malarial fevers, and caused by the co-existence of the two pathogenic factors, which separately produce typhoid and malarial fevers, then it would seem to be difficult to find a more appropriate name than "typho-malarial fever."

Dr. Eugene Palmer has a classical paper on the "History and Mechanism, or Modus Operandi of Anæsthetics," in which he reviews the early experiments with ether and nitrous oxide, and lays down some good rules for meeting accidents from the administration of anæsthetics. This paper is worth reading.

Dr. C. F. Paine, of Commanche, gives the history of a very remarkable case of hæmatemesis, occurring in a stout man, of 60, and which was attended with convulsions. The man died. The cause was obscure, but like many such cases, was supposed to be due to an enlarged liver or spleen, or both. Patient was a hard worker, addicted to strong drink.

A very extraordinary case is reported by Drs. Bowers, Coleman and Lancaster, under the comprehensive title, "Typhlitis, Peri-typhlitis, Para-typhlitis and Spleenitis." A child, aged 29 months, fed on acorns until there was a distension of the whole abdomen, inflammation, and an agglutination of all the internal organs. The only wonder is that the child did not succumb to the violence of the inflammation.—which must have been violent to result in such extensive adhesions. Was this not probably a case of impaction of the whole colon?

Dr. T. J. Bennett relates some interesting experiments which go to show the antagonism between chloral and strychnia, and between strychnia and urethan, which led him to believe that urethan would control spasms from any cause, and to try it; which he did, successfully, in a case of puerperal convulsions. This is a valuable suggestion.

Dr. S. T. Lowry gives an account of a case of dermatitis, with complete desquamation, produced by the internal administration of quinine.

In the section of obstetrics Dr. H. A. West's paper on placenta prævia was pronounced by a member "the most classical paper he had seen." It is a good paper, worthy of a careful perusal. At the election of officers Dr. West was complimented with the chairmanship of the section on Medical Jurisprudence, Chemistry and Psychology. A good selection.

Dr. E. J. Beall, of Fort Worth, relates the diagnosis and removal of an interstitial fibroid, in a parturient woman. The tumor was enucleated, and the child delivered successfully; mother and child both survived. This paper is illustrated with a cut, showing the relative position of tumor and child. It was prepared originally for this JOURNAL—and it was an after-thought to read it at the Association meeting. The paper appeared in our May number.

Dr. J. D. Osborn's paper on "Puerperal Eclampsia, Treated by Verat. Veride Hypodermically," is good reading. The Doctor gets best results from that method. The reading of this paper elicited a lively discussion, and brought out a great diversity of views, and modes of treatment.

Dr. Osborn's address as Chairman of the Surgical Section, "Antisepsis," is a masterly review of the subject, interspersed with illustrations of the beneficent results of antisepsis in his hands.

In 39 years practice Dr. S. Farrow Styles met six shoulder presentations in 2000 deliveries. He gives the history of each;—a valuable contribution to the statistics of midwifery.

Dr. S. H. Stout, one of the "fathers," who will be remembered by all old Confederate surgeons as the very efficient Medical Director of the Hospital Department of the Army of Tennessee,—subsequently Professor of Obstetrics in Atlanta Medical College—treats of the "Management of the Third Stage of Labor," from the standpoint of personal experience, and lays down some excellent precepts.

Dr. J. P. Oliver relates the details of a "Lithotomy for Supposed Encysted Calculus of the Bladder, with many Ugly Complications." "When the cavity of the bladder was thoroughly explored, * * no calculus was was found, but instead, several tumors about the size of a large buckshot at the site where the supposed calculus was invariably felt; nor could the sound be made to locate the rough substance we had so forcibly felt a few minutes previously. * * * I am still inclined to the opinion that the little tumors may have been small calculi imbedded, covered with phosphatic deposits." Patient died two months and eleven days after the operation. A very interesting paper.

"A case of Conservative Surgery," by Dr. J. D. Bass, is a valuable lesson. The Doctor had a badly torn arm, in which much tissue was lost. He attempted to fill in with sponge, as Drs. Beall & Adams had been successful in grafting, but it was disastrous. He says the sponge becomes a source of constant irritation, and keeps up suppuration. The attempt was abandoned, and the arm finally amputated. This report may prevent some one from trying the sponge to the exclusion of known and tried means which promise better results.

One of the most creditable papers, as well as operations, is that by Dr. John C. Jones, of Gonzales. Dr. Jones operated successfully for a strangulated gut (small intestine encircled by a peritoneal band), in a man of 70, after eleven days strangulation. The operation did not last over forty minutes. Patient was a subject of inguinal hernia, and the hernia became constricted after being reduced.

Dr. Luther B. Creath contributes a well considered paper on "Scrofulous Glands of the Neck, and Their Removal." The paper shows much reading and much sensible reflection, and is one of real merit. We commend it to our readers, and especially to young physicians.

Dr. E. J. Ward, one of the most fluent writers in the association relates how he ligated the popliteal artery for a case of elephantiasis arabum.

Dr. F. H. Tucker, relates the very remarkable occurrence of a congenital imperforate prepuce, in a child of five months. The mother stated that the child had passed no water since birth: but the doctor says, there was a recto-vesical fistula, through which the urine escaped. Relief by circumcision.

Dr. Tucker, also, as chairman of the section on Gynecology contributes a good paper on "Pelvic Hæmatoma", in which he reviews the work in gynecology in the past twelve months.

Dr. L. J. Graham makes a good showing of obstetric skill and learning in his "Report of a few Obstetric Cases."

Dr. C. F. Paine, relates a Craniotomy and comments on Craniotomy in general.

One of the most remarkable, as well as interesting papers, is that by Dr. J. W. Carhart in which he tells how he treated (successfully) a large spina-bifida, which was ruptured during delivery.

He says: "There was a sudden gush of water, larger in quantity than on the rupture of the membranes, and I felt the release of the child as from a vise, and it was born. The first glance at the back, revealed the cause of the obstruction. The emptied sac, which, when full of fluid must have been as large as the child's head, lay folded back over the childs buttocks, revealing a bleeding surface from which the tumor had been torn, in the lumbo-sacral region, about three inches in diameter. * * * "I cut out of the empty sack a flap of skin of requisite size and shape to completely cover the naked spot between the hips from which the tumor was torn.

This flap was of course, adherent for one-third of its circumference at the lower segment.

After washing the parts with warm bichloride solution of about 1-5000, I dusted them lightly with powdered iodoform, and, bringing the edges nicely in apposition, I sewed them with fine silk, taking about fourteen continuous sutures. I applied adhesive straps, in various directions, over the whole, gave it to the nurse for a very limited ablution and ordered it kept on its face, with head slightly lower than the hips, and gave no internal medication, as it was not indicated, and ordered no more handling than was strictly necessary."

Dr. O. J. Kendall tells of a case in which a negro woman gave birth to a pair of "assorted" twins, one white and one black.

Dr. P. J. Bowers relates his difficulties with a "Complicated Case of Labor," breech presentation, complicated by partial placenta prævia, adherent at that, in a woman who had previously given birth to five children, and each labor was characterized by adherent placenta.

In the section on Ophthalmology the chairman Dr. T. J. Tyner, of Austin, read his address, a paper in which he briefly, but clearly reviewed the progress and dcings in that interesting and important branch of Medical Science, discusses the value of certain drugs, and compares the various modes of operation, etc. With regard to the so-called-new-treatment after cataract operation, (by adhesive strips, instead of the bandage, claimed recently by Dr. Chisholm, to have originated with him in the Presbyterian Hospital in Baltimore), Dr. Tyner had practiced it for years, having learned it of DeWecker, in Paris ;—but the Doctor says : "I now, in nearly every case, apply the bandage the first, and sometimes for the second night, [as a protection from injury, and not for the darkness], changing it for the plaster the next morning. This rule I invariably adopt with the Mexicans, of whom I had many under treatment during my residence in San Antonio. It may not be out of place to state here, as I have had occasion to do before the West Texas Medical Society, that in every case of extraction in that class of people, I have had the most troublesome conjunctivitis, commencing about the sixth to the eighth day, and lasting from four to six weeks. Treatment seemed to do no good; after a given time the inflammatory symptoms would subside, and a good recovery was had in every case."

The reason why Mexicans are more liable to conjunctivitis after extraction, the doctor thinks,—and he is the first, we believe, to point out this clinical fact,—is because of their habits of life. He says," for the most part they are very poor, live in uncomfortable houses, and upon the poorest and scantiest food." "This inflammatory tendency," says the doctor, "was in direct contrast to the whites—in whom inflammatory reaction was the exception."

Dr. R. H. Chilton of Dallas has a good paper on "Iritis,"—a paper which shows that the doctor is an enthusiast in his chosen field, and knows how to handle the pen as well as the scalpel.

Dr. J. H. Smith, relates a case of Neuro-Retinitis in a German woman, in which the value of a knowledge of the subject, and of medical skill was demonstrated; a creditable case for the doctor.

The report of the chairman of the section on Electro-Therapeutics—the venerable Dr. F. T. Paine, reads like a work of fiction, so full is it of rare cases. and most unusual results. The Doctor has devoted his time wholly, for three years, to observing the effects of electrical treatment, and has tried it in all sorts of cases; and the result, as related in the admirable paper before us, is astounding. It would seem to open up a new field for experimental research, and that there is scarcely a limit to the possibilities of this wonderful agent, guided and directed by intelligence, and the ripe experience of three score years and ten. If future observation shall confirm the statements here recorded by Dr. Paine, there is no estimating the value of electricity as a remedial agent. We wish we had the space to give some of his cases, and especially, to describe his peculiar views as to the existence of an "electrical anæsthesia" (he calls it;) in a large number of cases. Dr. Paine says, that there are women whose vagina and other parts are insensible to an electrode, which if applied to another, would seem like a red hot iron. We advise our readers to study this paper; it is full of interest. By the bye, we are proud to see that the *British Medical Journal* makes mention of Dr. Paine's paper on *Solix Nigre*, published in Transactions for '84, the first Texas paper ever quoted by that Journal, so far as we know.

Under this section Dr. R. G. Williams has a good paper on

Rheumatism, Neuralgia and Vertigo, treated by the electric current.

The book closes with the Section of Gynecology, in which there are only two papers, one by the Chairman, Dr. F. H. Tucker, of San Augustine—to which we have alluded, when mentioning his other papers—and one, the last in the series—by Dr. B. E. Hadra, of Austin, entitled, "The Difficulties and Shortcomings of Trachelorrhaphy, and Some Suggestions How they may be Overcome," a paper written in the usual forcible style of this well known author. Dr. Hadra is remarkable for his originality. He has devisid several operations, or rather, modified certain recognized procedures, and his manner of operating is well known to his *confreres*. Dr. Hadra has a book now in press on the subject of the female perineum—"Lesions of the Pelvic Floor," it is called, and which we hope soon to have the pleasure of reviewing.

The section work is followed by Dr. Wooten's address on "The University of Texas." As this paper has appeared in full in the JOURNAL, we will not speak of it here.

The Appendix contains the roll of members, officers since organization, Code of Ethics, Constitution and By-Laws, resolutions, ordinances, etc., and an index.

The printing was done by Eugene Von Boeckmann, of Austin, and the binding by John Southgate. There are 440 closely printed pages, long primer, and 39 lines to the page, and 468 words. It was done at the astonishingly low price of $1 per page, and cost, therefore, not exceeding 75 or 80 cents per copy. There was one-third more manuscript than last year, and had it been printed in same type, would have made 880 pages, or one-third larger than the large volume of 1886. The presswork and paper are good, and we think it a most creditable work, especially considering the cost. We are pleased to say the work has met the hearty approbation of President Burroughs, who is kind enough to compliment the Secretary and the Publishing Committee in the following graceful language:

"Please allow me to congratulate the Committee on Publication for the elegant, admirable and efficient manner in which it has discharged its duty in presenting the members with such a handsome volume of Transactions. Too much praise cannot be awarded to the artistic and scientific selection and arrangement of the articles comprising the volume."

[And to the Secretary.]

"I think your work has been admirably executed in the preparation of the Minutes, and in the publication and issue of the Transactions."

And our esteemed Treasurer,—the "old faithful"—as some call him, expresses himself on the subject in his blunt, but kind way as follows :

"Transactions first rate : everybody ought to be happy"—while Dr. Geo. Duffield the courteous Secretary of the Michigan Medical Association writes :

"Dear Doctor Daniel. Permit me to again thank you for the excellent Transactions of your energetic State Medical Society. They are excellent, and full of interesting papers from beginning to end. Accept my warmest thanks."

We are forced to the conclusion that in future we should have more money or fewer papers—less manuscript. This work is large enough, and valuable enough to warrant a handsome cloth cover, but it was by the closest economy only, and by competitive bids that the Committee was enabled to get it published even in so good a style, with the means at their command.

